# PKAN hiPS-Derived Astrocytes Show Impairment of Endosomal Trafficking: A Potential Mechanism Underlying Iron Accumulation

**DOI:** 10.3389/fncel.2022.878103

**Published:** 2022-06-16

**Authors:** Maddalena Ripamonti, Paolo Santambrogio, Gabriella Racchetti, Anna Cozzi, Ivano Di Meo, Valeria Tiranti, Sonia Levi

**Affiliations:** ^1^Vita-Salute San Raffaele University, Milan, Italy; ^2^Proteomics of Iron Metabolism Unit, Division of Neuroscience, IRCCS San Raffaele Scientific Institute, Milan, Italy; ^3^Psychiatry and Clinical Psychobiology, Division of Neuroscience, IRCCS San Raffaele Scientific Institute, Milan, Italy; ^4^Unit of Medical Genetics and Neurogenetics, Fondazione IRCCS Istituto Neurologico Carlo Besta, Milan, Italy

**Keywords:** PKAN disease, endosomal trafficking, cellular iron uptake, iron accumulation, neurodegeneration

## Abstract

PKAN disease is caused by mutations in the *PANK2* gene, encoding the mitochondrial enzyme pantothenate kinase 2, catalyzing the first and key reaction in Coenzyme A (CoA) biosynthetic process. This disorder is characterized by progressive neurodegeneration and excessive iron deposition in the brain. The pathogenic mechanisms of PKAN are still unclear, and the available therapies are only symptomatic. Although iron accumulation is a hallmark of PKAN, its relationship with CoA dysfunction is not clear. We have previously developed hiPS-derived astrocytes from PKAN patients showing iron overload, thus recapitulating the human phenotype. In this work, we demonstrated that PKAN astrocytes presented an increase in transferrin uptake, a key route for cellular iron intake *via* transferrin receptor-mediated endocytosis of transferrin-bound iron. Investigation of constitutive exo-endocytosis and vesicular dynamics, exploiting the activity-enriching biosensor SynaptoZip, led to the finding of a general impairment in the constitutive endosomal trafficking in PKAN astrocytes. CoA and 4-phenylbutyric acid treatments were found to be effective in partially rescuing the aberrant vesicular behavior and iron intake. Our results demonstrate that the impairment of CoA biosynthesis could interfere with pivotal intracellular mechanisms involved in membrane fusions and vesicular trafficking, leading to an aberrant transferrin receptor-mediated iron uptake.

## Introduction

PANK-associated neurodegeneration (PKAN, OMIM *606157) disorder belongs to a heterogeneous group of neurodegenerative diseases, named Neurodegeneration with Brain Iron Accumulation (NBIA), that presents huge iron overload in specific brain regions and extrapyramidal dysfunction as a common denominator (Levi and Tiranti, 2019). PKAN approximately accounts for 50% of NBIA cases, it is characterized by a striking phenotype, presenting with early-onset spastic-dystonic features with a later appearance of Parkinsonian features, cognitive impairment, obsessive-compulsive disorder, and brain iron accumulation (Hogarth et al., 2017). Generally, PKAN reveals in childhood with gait troubles and quickly degenerates to severe movement deficits coupled with dystonia, dysarthria, and dysphagia. The trademark of PKAN is a particular feature called the “eye-of-the-tiger” sign observable in the globus pallidus in T2-weighted MRI (Zorzi et al., 2011). PKAN is caused by mutations in the *PANK2* gene (Zhou et al., 2001), which codifies for one of the four known isoforms of Pantothenate Kinase enzyme (PANK1 and 1β, PANK2, PANK3, and the inactive PANK4) (Hayflick et al., 2003; Liu et al., 2019; Yao et al., 2019). PANK2 is particularly abundant in brain tissue (Zhou et al., 2001) and catalyzes the first limiting step of the chain reactions that lead to CoA biosynthesis. Human PANK2, unlike other cytosolic isoforms, is specifically localized in mitochondria (Hörtnagel et al., 2003; Kotzbauer et al., 2005; Johnson et al., 2006). Despite several crucial findings that have been obtained in the last years, neither the mechanisms that lead to iron accumulation nor the correlation between hypothetical defective mitochondrial CoA amounts and PKAN pathogenesis has been fully understood and no resolutive cure is available. Several animal and cellular models have been developed trying to clarify the molecular events that generate this disorder (Kuo et al., 2007; Brunetti et al., 2012, 2014; Orellana et al., 2016), showing various pathological phenotypes common to humans but lacking the severe iron accumulation. We exploited a cellular model based on human hiPS-derived astrocytes from PKAN patients, which showed iron accumulation during several *in vitro* passages mimicking the aging process (Santambrogio et al., 2022). Astrocytes serve critical functions for the maintenance and protection of neurons and participate in neurophysiological processes as, for example, synaptic transmission (Kimelberg, 2010). In particular, they are responsible for iron buffering in brain tissue functionality and for neuronal protection against iron toxicity due to the production of deadly reactive oxygen species (ROS) (Bylicky et al., 2018). A fine regulated cellular iron entry route is Transferrin Receptor 1 (TfR1)-mediated endocytosis of Transferrin (Tf)-bound iron; subsequent iron release from Tf occurs in the early endosomal pathway thanks to the acid lumen of endosomes (Dautry-Varsat, 1986; Aisen, 2004), and TfR1 is then recycled back to the plasma membrane by specific compartments called recycling endosomes (Maxfield and McGraw, 2004). There are evidence demonstrating that direct transient interactions between cargo endosomes present in the cytosol and containing Tf-bound iron and mitochondria are instrumental for iron transfer between these two organelles (Das et al., 2016; Hamdi et al., 2016). Moreover, iron-mediated alterations in cargo vesicles could impair these interactions, eventually misleading a correct iron delivery (Das et al., 2016). In fibroblasts from different NBIA sub-types, an impairment in TfR1 palmitoylation results in a defective TfR1 recycling, leading to intracellular iron accumulation (Drecourt et al., 2018). In addition, it was reported that an altered lipid composition of red blood cells plasma membrane of PKAN patients, in particular, an increase in sphingomyelin/phosphatidylcholine and sphingomyelin/phosphatidylethanolamine ratios, was associated with membrane fluidity distress (Aoun et al., 2017). Thus, a potential alteration in endosomal and plasma membrane lipid composition could affect vesicle exo-endocytosis, organelles fusions, and also intracellular trafficking in astrocytes, leading to an impaired TfR1-mediated iron intake and its accumulation over time.

In this study, we employed the activity-enriching marker SynaptoZip (Ferro et al., 2017) to investigate constitutive exo-endocytosis and vesicular dynamics in PKAN-derived astrocytes. This tool is based on an engineered VAMP2 protein that carries, at the intraluminal side, a “bait” for a small cognate fluorescent peptide called Synbond. By expressing this protein, called SynaptoZip, it is possible to irreversibly tag each vesicle that has undergone exo-endocytosis to estimate the activity of individual endosomes by cumulative fluorescence of Synbond-enriched vesicles. We found that PKAN astrocytes have an impaired endosomal compartment functioning as compared to controls. Interestingly, this anomalous phenotype is partially rescued after acute pharmacological treatments able to counteract impaired CoA biosynthetic pathway typical of PKAN. Based on our results, we hypothesized that a CoA-dependent impairment of vesicular trafficking could lead to a detrimental iron accumulation in astrocytes, which might trigger a cascade of events responsible for neuronal death in PKAN patients.

## Materials and Methods

### Generation of Astrocytes From Human iPSC

PKAN and control astrocytes were prepared as detailed in Santambrogio et al. (2022). In brief, hiPS clones for each control and PKAN patient were obtained and characterized as previously reported (Orellana et al., 2016). hiPS clones from control and PKAN patients were then differentiated into pure population of Neuornal Precursor Cells (NPCs) as previously described (Orellana et al., 2016). NPCs were plated onto Matrigel-coated dishes and maintained in DMEM-F12 supplemented with 1% Pen/Strep (Sigma Aldrich, St. Louis, United States), N2 (1:100, Life Technologies, Carlsbad, United States), B27 (1:200, Life Technologies, Carlsbad, United States), 2 mM L-glutamine (Sigma Aldrich, St. Louis, United States), and hFGF (20 ng/ml, Tebu-Bio Italy, Magenta, Italy). As the NPCs culture reached 60% confluence, 20% heat-inactivated (56°C, 30 min) FBS was added to the culture media and the culture produced mature astrocytes. The culture was maintained for 30 days before checking maturation (Supplementary Figure 1).

### Immunoblotting and Immunostaining

Immunoblotting was performed after the separation of soluble proteins by SDS-PAGE as described in Orellana et al. (2016). The signal was revealed using an ECL kit (GE Healthcare, Chicago, United States) and collected by ChemiDoc MP Imaging System (BIORAD, Hercules, United States) (Abs: mouse TfR1, Zymed Laboratories, San Francisco, United States Cat #13-6800, 1:1,500; mouse β-actin, Sigma Aldrich, St. Louis, United States Cat #A5441, 1:600). Cells grown on coverslips were fixed in 4% paraformaldehyde immunofluorescence was done as in Santambrogio et al. (2020). Specific primary antibodies used were: rabbit anti-GFAP (Glial Fibrillary Acidic Protein Agilent Technologies Italia, MIlan, Italia Cat# GA524, RRID:AB_2811722, 1:250 dilution) and mouse anti-EAAT2 (Excitatory Amino Acid Transporter 2, Santa Cruz Biotechnology, Dallas, United States Cat# sc-365634, RRID:AB_10844832). Specific Donkey secondary antibodies Alexa-488 and Alexa-546 conjugated were used (#IS20015, #IS20305, 1:800 dilution, Immunological Sciences, Rome, Italy). Images were acquired by fluorescence microscope Zeiss Axio Observer.Z1 equipped with Hamamatsu EM-CCD 9100-02 camera and Volocity acquisition software.

### Transferrin and Synbond Live Uptake

Lentiviral vectors encoding SynaptoZip (SZ) were prepared and used as described (Ferro et al., 2017) to infect 35 days old astrocytes. Pulse and chase experiments with fluorescent transferrin and Synbond (SB) were performed on 50 to 60 days old astrocytes.

#### Pulse and Chase With Transferrin

A total of 50–60 days old astrocytes were starved in serum-free media for 1 h before incubation with 25 μg/ml Alexa-546-Transferrin or Alexa-647-Transferrin (Tf) (Invitrogen, Waltham, United States, #T23364, #T23366), 5 or 15 min, 37°C, and 5% CO2 in serum-free F12 DMEM (Lonza Group, Basel, Switzerland, #12-719F). After incubation cells were washed in 4°C PBS, Euroclone, Milan, Italy + 1% BSA, Applichem GmbH, Darmstadt, Germany they are rinsed in 4°C PBS, fixed in 4% paraformaldehyde, and mounted for retrospective fluorescence analysis. Fluorescence was acquired by confocal imaging by LEICA, Wetzlar, Germany TCS SP5 laser scanning microscope equipped with proper emission/absorption filters. Laser parameters were set to not saturate pixels, and images were acquired at 1,024 × 1,024 or 2,048 × 2,048 resolution, 12 bits, with 40x 1.25 NA or 63X 1.4NA oil immersion objectives. Laser power, gain, contrast, and all the acquisition parameters were kept constant during the same experiment. Analyses were run as follows: Fiji software (Fiji, RRID:SCR_002285) was used for image analyses; the mean Tf fluorescence for each cell was calculated and analyzed, and results were presented as normalized on controls. Cell borders were depicted exploiting SZ fluorescence, and ROIs were generated and the mean fluorescence on the Tf channel was measured. All the fluorescence analyses were run on raw images, and the mean filtering and subsequent LUT transformation (by ImageJ built-in LUTs) with the same parameters for all images was applied (the subject is LUT transformation) only for the presented Figures, just for representative purposes to better appreciate differences in fluorescence intensities by eye.

#### Pulse and Chase With Synbond

The 50 to 60 days astrocytes expressing SZ were live incubated with 5 nM SB-Alexa-647, 5 or 15 min, 37°C, and 5% CO2 in serum-free DMEM F12. After incubation cells were washed in 4°C PBS + 1% BSA, rinsed in 4°C PBS, and fixed with 4% paraformaldehyde, and mounted for retrospective fluorescence analysis. For double epoch experiments (Figures 2F–I) after cold wash with PBS + 1% BSA, the same samples were incubated with 5 nM SB-Alexa-568 for the same time pulse and in the same conditions as the first one, then washed again with 4°C PBS + 1% BSA, rinsed in 4°C PBS, fixed in 4% paraformaldehyde, and mounted for retrospective fluorescence analysis. (SB peptides were produced by JPT Peptide Technologies GmbH, Berlin, Germany). Images for retrospective fluorescence analyses were collected as described above. Mean single vesicle fluorescence was calculated for each image, and segmentation of vesicles was run in a semi-automated way on the SZ signal. Puncta were segmented based on classic watershed transform and Voronoi tessellation, exploiting MATLAB (MATLAB, RRID:SCR_001622, Mathworks, Natick, United States) standard built-in functions/plugins, as previously described (Ferro et al., 2017). An inclusion/exclusion fluorescence threshold was set for identified *Bona Fide* vesicles as follows: background SZ fluorescence was calculated from vesicle devoid regions and the threshold for inclusion was set as mean background fluorescence + 2 sd. The so obtained ROIs were used to calculate the mean fluorescence of individual vesicles, on each channel, as follows: SB signal was first normalized over SZ fluorescence for each vesicle, then obtained average results from each condition were normalized over controls proper of each experiment. A single endosome was considered active when its mean SB fluorescence was higher than the mean background SB fluorescence + 2 sd. Background SB fluorescence was calculated from vesicle devoid regions. For dynamic binding experiments (Figure 2E), live astrocytes expressing SZ were mounted on a GE Healthcare Deltavision™ Ultra microscope equipped with a cage incubator, CMOS camera, led light lines, and proper emission filters. Cells were maintained for the time of the experiment in DMEM F12 serum-free medium and 5 nM SB was added directly into the acquisition chamber. Before adding SB, some fields of interest were selected and then the automated built-in navigation system was used to switch the acquisition among these fields. SB fluorescence was live-followed starting from 1 min after application (T_0_) up to 15 min capturing a z-stack image of the selected fields every minute (40x 1.35NA oil immersion objective, 1,020 × 1,020 px). Collected images were deconvoluted by DV SoftWorX software (SoftWoRx software, RRID:SCR_019157) and the SB fluorescence was analyzed. At least five cells for each condition were analyzed by ImageJ software as follows: *n* = 5 ROIs were manually generated for each cell, and mean SB fluorescence was calculated for each time point. T_0_ average value was used to normalize the uptake trend over time. A mean value and relative SD, from three consecutive time points, from each ROI, from each condition, were calculated and average means were plotted as in Figure 2E.

#### Vesicular Tracking

The 50–60 days astrocytes expressing SZ were live incubated with 5 nM SB-Alexa-647, 37°C, and 5% CO2 in serum-free DMEM F12, washed with DMEM F12 + 1% BSA, and mounted on a GE Healthcare Deltavision™ Ultra microscope equipped as mentioned before. Cells were maintained in a serum-free DMEM F12, 37°C, and 5% CO2 for the entire experimental time. SB fluorescence was live imaged at high resolution (60x oil immersion) at 20 fps, and this recording speed was the fastest possible, in our experimental setup, to maintain a good resolution and bit depth to distinguish individual vesicles and whole cells at the same time. Recordings with a good SNR and low bleaching were included in the analyses. Cells were analyzed as follows: ROIs from different cellular regions from the same cell were collected and SB-enriched vesicle trajectories were analyzed by Mosaic/Particle Analyzer 2D/3D ImageJ plugin (Sbalzarini and Koumoutsakos, 2005); data regarding MMS slope (MSSs), diffusion coefficients, and mean average distances were collected for each condition. For the statistical analysis presented in this study, only motile trajectories were considered. Motility was estimated by MSSs; all trajectories with an MSSs < 0.1 were considered as completely stationary objects and were not included. For diffusional behavior analyses, MSSs < 0.45 was considered as confined motion, 0.45 ≤ MSSs < 0.55 as diffusive, and 0.55 ≤ MSSs ≤ 1 as directed motion.

### CoA and 4-PBA Treatments

Astrocytes were differentiated and maintained for 50–60 days in 20% heat-inactivated FBS, single doses of CoA 25 μM, 3 days (Sigma Aldrich, St. Louis, United States, Cat #C4780) and 4-phenylbutyric acid (4-PBA) 0.5 μM, 24 h (Sigma Aldrich, St. Louis, United States, Cat #P21005) were directly added in the culture medium. In these conditions (heat-inactivation 56°C, 30 min), the serum enzymes responsible for the quick degradation of CoA are inactivated, as demonstrated in Srinivasan et al. (2015). Cells were switched in serum-free media only for the short timeframes of pulse and chase or live-tracking experiments.

After addition of CoA and 4-PBA, cells were maintained in 5% CO2, 37°C for the treatment times, and then culture media was changed based on the programmed experiment (see above).

### Statistical Analysis and Figures

Statistical tests to predetermine the sample size in the experiments were not used. Statistical analysis and graphs generation were run in GraphPad Prism 9 (GraphPad Prism, RRID:SCR_002798, San DIego, United States), statistical tests used were Student’s *t*-test, Mann–Whitney test, KS test, and One-Way ANOVA as detailed in the text, Figure legends, and Tables. Figures and schemes were arranged by Serif Affinity Designer 1.10 software (Serif Europe, West Bridgford, United Kingdom). All experiments were replicated at least three times and normalized as detailed in the text, Figure legends, and Tables. A *p*-value < 0.05 was considered statistically significant (*p < 0.05; ^**^p < 0.01; ^***^p < 0.001; ^****^p < 0.0001).

## Results

### PKAN Astrocytes Showed an Increased Transferrin Uptake

**FIGURE 1:**
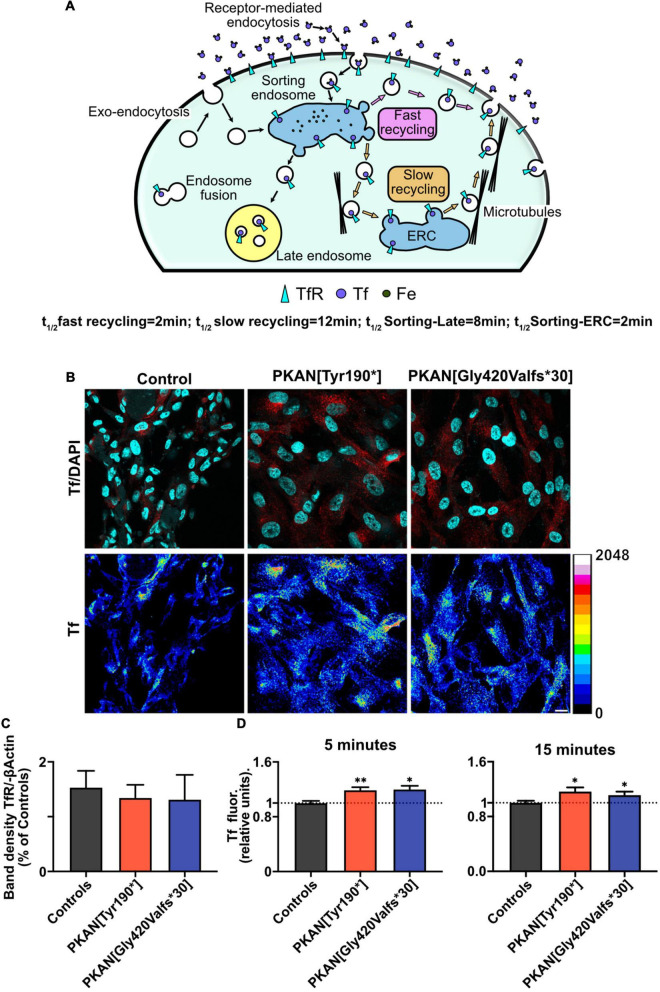
PKAN astrocytes show an increased Tf uptake. **(A)** Schematic cartoon depicting the structure and functioning of the main endosomal compartments fate and vesicular tagging by Tf. **(B)** Confocal images of astrocytes, and their live uptake of fluorescent transferrin applied for 15 min (Tf red in upper panels, and 16 colors LUT, as indicated alongside, in lower panels), in controls and two different PKAN patients [PKAN(TYR190*); PKAN[Gly420Valfs*30)] (scale bar 20 μm). **(C)** Quantification of Western blot analyses for TfR1 (ns, One-Way ANOVA, *n* = 3 independent replicates). **(D)** Histograms show the mean cellular Tf uptake in astrocytes from control and PKAN patients at different pulse and chase time (Mann–Whitney test, *n* ≥ 95 cells analyzed for each, from at least three independent replicates). Error bars are SEM for all. *p < 0.05; ^**^p < 0.01; ^***^p < 0.001; ^****^p < 0.0001 for all.

Astrocytes were differentiated from previously obtained hiPSC and characterized as previously described (Santambrogio et al., 2022; Supplementary Figure 1). These hiPS-clones (Orellana et al., 2016) originated from three neonatal normal subjects, here called controls, and two PKAN patients, one carrying a c.[569_570insA] homozygous mutation leading to the expression of a protein with a premature stop codon and one a c.[1259delG] homozygous mutation leading to the expression of a protein with a frameshift, respectively (see Table 1 for details) (Santambrogio et al., 2015; Orellana et al., 2016). Both mutations result in the complete absence of the PANK2 enzyme (Santambrogio et al., 2015). Experiments were run on cells after 50–60 days from the beginning of differentiation from NPCs. At this early stage (young), PKAN astrocytes do not show detectable iron accumulation. Moreover, no evidence of significant overexpression of TfR1, compared to controls, emerged by Western blot analyses (Figure 1C and Supplementary Figure 2A). Thus, the analysis of young astrocytes could allow dissecting the early mechanisms that lead to iron burden in cells older than 70 days (Santambrogio et al., 2022). The post-endocytic fate of TfR1 has been well-characterized, and it can be used to identify different classes of endosomes based on its enrichment after different incubation time points (Lin et al., 2004; Drecourt et al., 2018) as resumed in Figure 1A. We performed pulse and chase experiments in which astrocytes, from controls and PKAN patients, were live-incubated with fluorescent Tf (Alexa-546-Tf or Alexa-647-Tf) at given time points to selectively label different endosomal compartments. As shown in Figures 1B,D and Supplementary Figure 2B, at 5 and 15 min of incubation, there is a significant increase of Tf uptake in both PKAN young astrocytes as compared to controls, thus suggesting the hypothesis of a potential alteration of the TfR1 recycling pathway. Since no alteration in the expression levels of TfR1 was seen (Figure 1C and Supplementary Figure 2A), this increased incorporation of Tf, in young astrocytes, could represent a potential mechanism for the iron overload and subsequent accumulation previously seen in older cells (Santambrogio et al., 2022).

**TABLE 1:** PKAN astrocytes mutations.

Mutation	Protein	Here referred as
c.[569_570insA]	Stop codon p.[Tyr190*]	PKAN[Tyr190*]
c.[1259delG]	Frameshift p.[Gly420Valfs*30]	PKAN[GlyValfs*30]

*Indicates stop codon.

**FIGURE 2:**
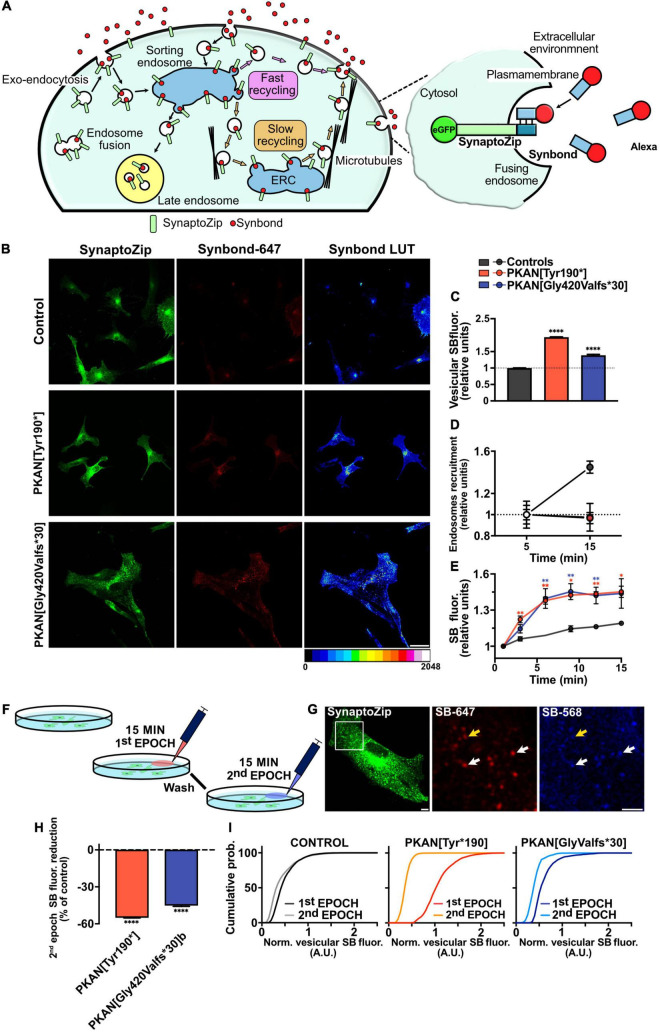
Constitutive Synbond uptake is impaired in PKAN astrocytes compared to controls. **(A)** Schematic cartoon depicting the structure and functioning of the main endosomal compartments’ fate and their vesicular tagging by Synbond, on the right is a detail depicting the basic functioning of the SynaptoZip-Synbond pair. **(B)** Confocal of astrocytes from controls and two different PKAN patients expressing SynaptoZip (green) and its Synbond live-uptake (red middle panels, and 16 color LUT right panels, as indicated on the bottom). Astrocytes expressing SynaptoZip were pulsed with 5 nM Synbond for 15 min to image the cumulative activity of the recycling compartment (scale bar 10 μm). **(C)** Histograms show the mean vesicular Synbond uptake in astrocytes from controls and PKAN (*t*-test). **(D)** Plots are the percentages of active endosomes, identified as described in methods and results, at different time points (5 min and 15 min) [**(C,D)** *n* ≥ 7,382 vesicles analyzed for each, from at least three independent replicates each]. **(E)** Binding dynamics from controls and PKAN astrocytes (Mann–Whitney test, *n* = 5 ROIs from *n* 5 cells analyzed for each, from at least three independent replicates each). **(F)** Schematic representation of the double pulse experiment to tag vesicles active during different epochs. **(G)** Confocal image of an exemplar astrocyte expressing SynaptoZip (green) and its Synbond vesicular uptake (red and blue) as in the protocol depicted in panel **(F)**. Middle and right panels are the regions indicated by the white square in the left panel. Synbond positive single endosomes are visible, some are enriched by both 647 and 568 Synbond peptides (white arrows) and others are positive for Synbond-647 but the fluorescence from Synbond-568 is faint (yellow arrow, scale bar 6 μm). **(H)** Bars are the mean vesicular percentages of Synbond fluorescence during the second epoch calculated on first epoch Synbond fluorescence, from the same vesicles, normalized on controls (one-way ANOVA). Curves in panel **(I)** are cumulative probabilities distributions of Synbond uptake in the two sequential epochs (15 min each), from controls and PKAN astrocytes, showing impairment in PKAN astrocytes uptake probabilities compared to controls [**(H,I)** *n* at least 800 vesicles analyzed for each, from *n* = 3 independent replicates]. Error bars are SEM for all. *p < 0.05; **p < 0.01; ***p < 0.001; ****p < 0.0001 for all.

### Constitutive Endosomal Exo-Endocytosis Cycle Is Impaired in PKAN Astrocytes

We investigated the activity and behavior of the constitutive endosomal trafficking, independently from receptor-mediated iron endocytosis, by using SynaptoZip-Synbond (SZ-SB) system (Figure 2A; Ferro et al., 2017). Thanks to this tool, it is possible to study the cumulative activity and the intracellular fate of single vesicles by analyzing the fluorescent Synbond (SB) peptide uptake (Ferro et al., 2017). This couple works as an activity marker generating a long-lasting fluorescent signal that can be acquired both live and retrospectively. The cumulative fluorescent signal results from the specific SynaptoZip-Synbond bound and subsequent entrapping of Synbond into an exocytosed vesicle. Synbond vesicle enriching relates to the number of exo-endocytic events and the average number of Synbond molecules that bind to available SynaptoZip at each fusion throughout the chosen experimental timeframe. In Figure 2B, we showed some representative young astrocytes expressing SZ (green) and their SB uptake (red and correspondent LUT transformation in right panels). No significant difference in viability and SZ expression was observed in PKAN astrocytes compared to controls (data not shown). To first investigate SB uptake, astrocytes expressing SZ were live incubated with SB and retrospectively analyzed by confocal microscopy. Single vesicles expressing SZ were segmented based on size and fluorescence, as described in the “Materials and Methods” section, and fluorescence for each SB-enriched endosome was analyzed; only endosomes with a SB fluorescence higher than the established threshold (see “Materials and Methods” section for details) were considered as active and included in the analysis (Figure 2C 15 min uptake and Supplementary Figures 3A,B 5 min uptake). PKAN astrocytes showed a significant increase of SB vesicular fluorescence compared to controls, indicating a general higher level of activity of constitutive trafficking of PKAN endosomes (Figure 2C 15 min SB incubation and Supplementary Figures 3A,B 5 min SB incubation). An estimate of endosomes “recruitment,” which is the fraction of active vesicles over time, was also obtained in astrocytes live pulsed with 5 nM SB for 5 and 15 min (Figure 2D and Supplementary Figure 5). Assuming this, with the SZ-SB pair, we can tag the entire endosomal compartment and detect the cumulative constitutive activity at the end of different epochs (5 and 15 min in this case), and in these conditions, an increase in cumulative activity is predictable as SB incubation time is augmented. However, while we observed a consistent increase in endosome recruitment in control astrocytes, this trend did not emerge in PKAN astrocytes. At first glance, these results appear to be in contrast with the evidence that PKAN endosomal compartment is more active at 15 min, but this increased activity is not due to an increment in vesicles recruitment. Therefore, we hypothesized that the same vesicle could be entrapped in a portion of aberrant plasma membrane, thus resulting in a different retention time affecting the normal endocytosis cycle. In this way, the vesicle trip toward its normal intracellular fate is severly impaired. To clarify this point, we followed SB uptake in living cells for 15 min to define SB-binding dynamics from PKAN and control astrocytes (Figure 2E and Supplementary Figure 3E). PKAN astrocytes showed faster SB binding dynamics compared to controls and apparently tend to saturate their available sites over the investigated time range (15 min). On the contrary, controls in the same timeframes still seem to follow a linear binding behavior (Figure 2E and Table 2). To corroborate this finding, we exploited two different SB peptides, conjugated with different fluorophores (Alexa-647, Synbond-647, Alexa-568, Synbond-568), to label endosomal compartment activity in two sequential epochs. The protocol is detailed in the “Materials and Methods” section and graphically resumed in Figure 2F. Since the SZ-SB bound is practically irreversible, it is possible to tag the same vesicle if it re-exposes to the plasma membrane in two different (Figure 2G) (Ferro et al., 2017). In this way, vesicles that are recycled back for a new exo-endocytosis cycle will be stained by both SB-tagged peptides (Synbond-647 and Synbond-568). Given the 1:1 SynaptoZip:Synbond binding ratio and the irreversibility of this bound, a similar amount of uptaken SB, and relative fluorescence, is expected to occur during the second epoch in the following cases: (i) a comparable number of exocytosis events of the same vesicle, (ii) a comparable exposure time of the same fusing vesicle to SB present in the extracellular environment, and (iii) a comparable availability of free binding sites for SB left from the first epoch. Vesicular fluorescence signals and their cumulative probability distributions were calculated from SB fluorescence in the two sequential epochs and the data obtained are presented in Figures 2H,I, respectively. As expected, considering known constitutive endosomal dynamics, a physiological reduction in SB uptake during the second epoch emerged in all cases (Supplementary Figure 3C,D), but a significant decrease of SB uptake during the second epoch emerged in PKAN astrocytes, if compared to the reduction seen in controls (Figure 2H). Cumulative functions of controls are almost completely overlapped, suggesting a similar ability, of the same endosome, to bind SB in the first and second epoch. On the contrary, PKAN endosomes cumulative functions are indicative of a significantly lower SB incorporation during the second epoch respect to the first, as predicted by the left shift of the curve seen in the second epoch (Figure 2I). PKAN endosomes reduced binding ability in the second epoch can be due to the higher amount of uptaken SB during the first epoch and the subsequent lower availability of SB binding sites during the second epoch respect to controls, as also suggested by binding dynamics (Figure 2E). This condition is also reinforced by the altered endosomal recruitment shown in Figure 2D and consequent higher exposure times or exocytosis events, for the same endosome, during the 1st epoch. Taken together, these data support the hypothesis of an impaired intracellular fate of endosomes in PKAN astrocytes, which lead to compromised exo-endocytosis cycles before being free to follow their natural routes.

**TABLE 2:** SB live binding dynamics.

	3 min	6 min	9 min	12 min	15 min
Controls	1.06 ±0.02	1.09 ±0.02	1.14 ±0.02	1.16 ±0.01	1.19 ±0.01
PKAN[Tyr190*]	1.22 ±0.03	1.38 ±0.03	1.42 ±0.02	1.43 ±0.02	1.45 ±0.05
PKAN[GlyValfs*30]	1.14 ±0.03	1.40 ±0.08	1.45 ±0.07	1.42 ±0.07	1.44 ±0.12
*p-value* PKAN[Tyr190*]	0.004	0.002	0.015	0.004	0.001
*p-value* PKAN[GlyValfs*30]	ns	0.005	0.001	0.002	ns

P-value was calculated for Controls vs. each PKAN patient; Mann–Whitney, n = 5 ROIs from n = 5 cells each. *Indicates stop codon.

### The Intracellular Fate of Endocytosed Vesicles Is Compromised in PKAN Astrocytes

**FIGURE 3:**
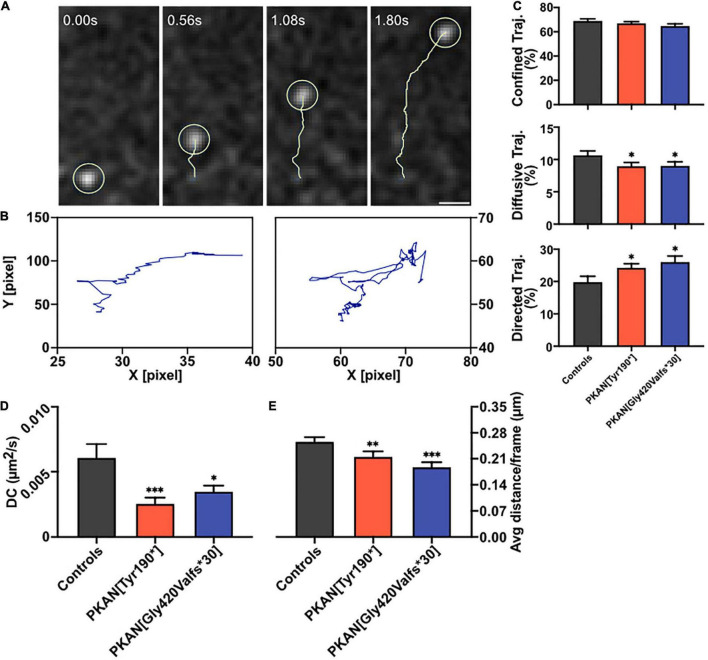
Endosomal dynamics. Astrocytes expressing SynaptoZip were pulsed with 5 nM Synbond for 5 min to tag only a*ctive early endosomes which undergone at least one exo-endocytosis cycle*. The fate of active endosomes was followed by high-speed live imaging and trajectories were analyzed. **(A)** An exemplar trajectory (yellow track) of a single endosome (light yellow circle, scale bar 1 μm). **(B)** Two exemplar trajectories represent a directed (left) and diffusive behavior (right). **(C)** Analysis of the diffusional behavior in control and PKAN astrocytes endosomes, bars are the mean percentage of trajectories that follows a confined (upper panel), diffusive, or directed (lower panel) type of motion. **(D)** Bars show the mean Diffusion Coefficient (DC) in astrocytes from control and PKAN in different conditions, calculated from dynamics of live-tracked endosomes enriched in Synbond. **(E)** Mean average distance covered by live-tracked endosomes in controls and PKAN patients [**(C–E)** statistical tests were run vs. controls, Mann–Whitney test, *n* ≥ 31 cells analyzed for each, from at least three independent replicates]. Error bars are SEM for all. *p < 0.05; ^**^p < 0.01; ^***^p < 0.001; ^****^p < 0.0001 for all.

To better understand the fate of an endocytosed endosome, cells expressing SZ were live-pulsed 5 min with SB to tag only active early endosomes which undergone at least one exocytosis cycle, then SB was washed, and fluorescence live-recorded at 20 fps. Feature point tracking of endosomes was then run on deconvoluted videos by the Mosaic/Particle Tracker 2D/3D ImageJ plugin (Sbalzarini and Koumoutsakos, 2005). From the mean square displacement (MSD) of the tracked particle, it is possible to dissect different characteristics of its motion such as diffusion coefficient (DC) and Moment Scaling Spectra slope (MSSs) (Ferrari et al., 2001; Sbalzarini and Koumoutsakos, 2005; Briane et al., 2018). Endosomes trafficking inside the cell follows biophysical laws and the aforementioned MSSs value is a good index to understand the type of motion and can be used to classify the behavior of endosomes (MSSs = 0 stationary object, MSSs < 0.5 confined motion, MSSs = 0.5 diffusive motion, 0.5 < MSSs < 1 directed motion). From a biological point of view, confined motion can represent an object that is moving in a closed region or in a wider but crowded area. The diffusive motion is the typical Brownian motion and can represent a free-floating endosome in the cytosol; instead, the directed motion represents an active motion that involves microtubular structures (Sbalzarini and Koumoutsakos, 2005; Briane et al., 2018). A representative tracked SB-enriched endosome is shown in Figure 3A (Supplementary Movies 1, 2), while in Figure 3B are shown exemplar trajectories from tracked endosomes with a directed (left) and diffusive (right) behavior. A generalized impairment of constitutive endosomal trafficking arose in PKAN astrocytes; in particular, a lower percentage of diffusive trajectories (Figure 3C middle panel), a higher percentage of directed trajectories (Figure 3C, lower panel), a lower diffusion coefficient (Figure 3D), and a lower average distance are covered by endosomes (Figure 3E) in PKAN astrocytes as compared to controls. The overall data are summarized in Table 3. These alterations agree with the hypothesis of an altered membrane exo-endocytic fusion process and, given the lower DC found, with a potential struggle for the endosome in moving along its physiological intracellular pathways. Likewise, the differences in directed motion and the lower average distance covered by motile endosomes in PKAN astrocytes are suggestive of a possible alteration in motor-driven motility along tubular pathways.

**TABLE 3:** Diffusional features of motile endosomes.

		%Confined	%Diffusive	% Directed	Avg. Distance μm	DC μm^2^/s
Controls	UT	69.0 ± 1.7	10.60.6	19.8 ± 1.8	0.26 ± 0.01	0.0060 ± 0.0010
	CoA	69.1 ± 1.5	11.40.7	18.5 ± 1.5	0.21 ± 0.01	0.0063 ± 0.0009
	4-PBA	66.4 ± 2.3	12.21.6	21.8 ± 2.7	0.23 ± 0.2	0.0051 ± 0.0014
PKAN[Tyr190*]	UT	67.0 ± 1.3	8.90.6	24.3 ± 1.2	0.21 ± 0.01	0.0025 ± 0.0004
	CoA	66.2 ± 1.4	11.11.6	24.7 ± 1.4	0.21 ± 0.01	0.0040 ± 0.0005
	4-PBA	65.9 ± 2.4	11.30.9	22.2 ± 2.3	0.22 ± 0.02	0.0041 ± 0.0008
PKAN[GlyValfs*30]	UT	66.5 ± 1.9	9.00.6	26.0 ± 1.9	0.19 ± 0.01	0.0034 ± 0.0004
	CoA	67.3 ± 1.0	9.30.5	23.8 ± 1	0.20 ± 0.01	0.0066 ± 0.0009
	4-PBA	69.9 ± 1.7	10.60.6	19.7 ± 1.5	0.19 ± 0.01	0.0064 ± 0.001

At least 7,000 motile trajectories analyzed for each condition. *Indicates stop codon.

### Acute Coenzyme A and 4-PBA Treatments Partially Rescued Aberrant Phenotype in PKAN Astrocytes

**FIGURE 4:**
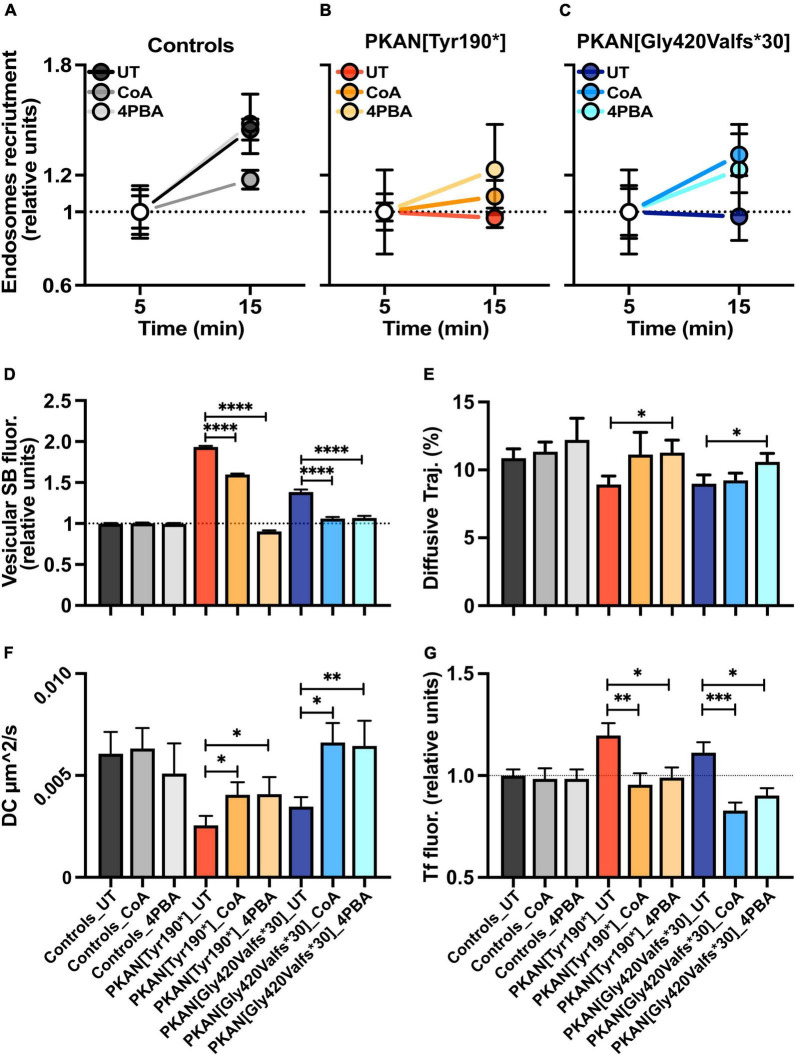
Effect of CoA and 4-PBA treatments on constitutive endosomal trafficking. **(A–C)** Plots are the percentages of active endosomes, identified as described in methods and results, at different time points (5 min and 15 min). **(D)** Bars show the mean vesicular Synbond uptake (15 min pulse) in astrocytes from controls and PKAN, treated with CoA or 4-PBA (*t*-test, *n* ≥ 5,706 vesicles analyzed for each, from *n* ≥ 3 independent replicates). **(E)** Mean % of diffusive trajectories in different conditions. **(F)** Mean Diffusion Coefficient in astrocytes from control and PKAN in different conditions, calculated from dynamics of live-tracked endosomes enriched in Synbond [**(E,F)** Mann–Whitney, *n* ≥ 20 cells analyzed for each, from at least three independent replicates]. **(G)** Plots are the Tf uptake (15 min) in controls and PKAN astrocytes treated with CoA or 4-PBA. In all cases, cells were treated with 25 μm CoA and 0.5 μm 4-PBA (Mann–Whitney, *n* ≥ 27 cells analyzed for each, from at least three independent replicates). Error bars are SEM for all. *p < 0.05; ^**^p < 0.01; ^***^p < 0.001; ^****^p < 0.0001 for all.

We then tested potential effects of acute treatments with CoA (72 h) and 4-PBA (24 h) on endosomal behavior. Direct treatments with CoA have been demonstrated to be efficient in rescuing aberrant PKAN behavior in hiPS-derived neurons and astrocytes (Orellana et al., 2016; Santambrogio et al., 2022). 4-PBA is an orphan drug with several properties, such as inhibition of pyruvate dehydrogenase kinase (PDK) that leads to an increase of activated pyruvate dehydrogenase complex (PDHC). PDHC catalyzes the oxidative decarboxylation of pyruvate to produce acetyl-CoA, which depends on mitochondrial CoA. It is plausible to hypothesize that the activated PDHC boosts, among others, acetyl-CoA levels, and lipogenic pathways in conditions of PDH deficiency (Ferriero and Brunetti-Pierri, 2013; Ferriero et al., 2013). Obtained data showed a partial rescue of endosomal trafficking and, for some features, 4-PBA was effective where CoA did not produce significant effects (Figure 4E). The failure in endosomal recruitment seen in PKAN astrocytes (Figure 2D) is partially restored by CoA and 4-PBA treatments. Indeed, an expected increase in endosome recruitment in PKAN astrocytes emerges only in treated conditions (Figures 4B,C). In control astrocytes treated with 4-PBA, a slight dampening of the endosomal recruitment trend also emerged, but this trend/effect is not statistically significant compared to UT condition (Figure 4A). Treatments also had an effect on the SB uptake in both PKAN patients, in particular, 4-PBA was able to bring back single vesicle SB uptake to the basal levels seen in controls (Figure 4D). CoA showed a similar ability on both PKAN patients, lowering the excess of SB uptake seen in UT conditions, restoring the endosomal activity to control values only in PKAN[GlyValfs*30] but not PKAN[Tyr190*] astrocytes, whose activity persisted as significantly higher than in controls. These data suggest that 4-PBA is more efficient in rescuing this feature, even in unhealthier conditions, such as the one found in PKAN[Tyr190*] (Figure 4D). CoA and 4-PBA also help in a partial rescue of some diffusional features of the endosomal compartment in treated astrocytes from both PKAN patients that showed a significant increase in DC compared to UT condition (Figure 4F). However, only 4-PBA proved to be efficient in restoring the percentage of diffusive trajectories toward values comparable to UT controls (Figure 4E and Table 3), while a similar, although not significant trend also emerged for directed trajectories (Table 3). The amelioration of global endosomal features carried out by CoA and 4-PBA treatments led to the restoration of correct Tf trafficking, significantly decreasing cellular Tf uptake in PKAN patients, and bringing back Tf uptake to levels comparable to UT controls in both PKAN patients (Figure 4G).

## Discussion

**FIGURE 5:**
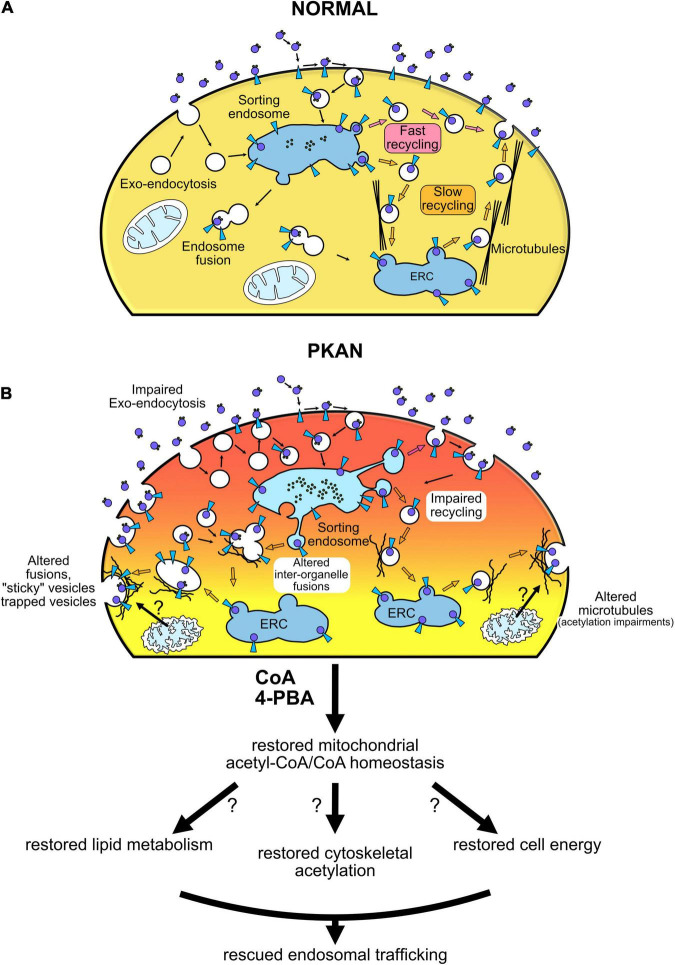
Vesicular trafficking impairment in PKAN leads to gliosis and neurodegeneration. Schematic cartoon illustrating our hypothesis of endosomal trafficking impairment in PKAN. **(A)** Normal condition, **(B)** altered dynamics in PKAN astrocytes. CoA deficiency in PKAN astrocytes leads to an impediment to normal physiological intracellular trafficking of the endosomal compartment. The aberrant endosomal behavior could be related to distorted biophysical properties of endosomes, such as membranes’ different lipid composition that modifies membranes fusion dynamics, but also to an unbefitting cytoskeletal milieu. The altered lipid composition by itself might also cause problems in the interactions between vesicles and normal cytoskeletal proteins.

TfR1-bound iron uptake occurs by clathrin-mediated endocytosis, then endocytosed vesicles follow internal pathways typical of constitutive endosomal trafficking (Maxfield and McGraw, 2004). A potential alteration in the exo-endocytosis process, in internal vesicle-organelle interactions (Das et al., 2016), as well as in the fate of endocytosed vesicles could potentially result in aberrant iron load that characterizes PKAN. Impaired iron homeostasis in astrocytes cause gliosis and trigger a cascade of events that can determine neuronal death (Rouault, 2013). Our data showed an increase in cellular Tf uptake in PKAN astrocytes compared to controls (Figures 1B,D). These results are compatible with a peculiar TfR1 recycling pathway malfunctioning, a behavior previously reported in fibroblasts derived from patients affected by different NBIA forms and by Friedreich’s ataxia, respectively (Drecourt et al., 2018; Petit et al., 2021). To investigate in deeper detail the fate of endocytosed vesicles independently from TfR1 pathway, we exploited the enriching-activity marker SynaptoZip (SZ) that allows the estimation of the exo-endocytosis activity from the cumulative fluorescence of cognate Synbond peptide (SB) (Ferro et al., 2017). We found an altered intracellular fate of endocytosed endosomes in PKAN astrocytes if compared to controls. We could therefore hypothesize that endocytosed vesicles could reside longer in an aberrant plasma membrane partition facilitating multiple fusion events, before letting the vesicles free to follow their own physiological intracellular fate. This hypothesis is strongly supported by our data, especially the finding that the augmented activity at 15 min is not due to an increase in vesicles employment, but to a higher “avidity” for SB of vesicles in PKAN astrocytes. Since SB fluorescence is normalized over SZ expression, and given that the SZ:SB binding ratio is 1:1 (Ferro et al., 2017), this behavior could fit only with a general increased exposure time of the vesicle to extracellular SB possibly due to multiple fusion events or to increased “stickiness” to plasma membrane of exocytosed vesicles. PKAN disease has been associated to an alteration in membrane lipid composition of red blood cells (Aoun et al., 2017). Moreover, there are evidences of lipid metabolism alterations in NBIA disorders in general (Kotzbauer et al., 2005; Aoun and Tiranti, 2015; Colombelli et al., 2015), which could explain the impaired membrane-membrane fusion. Another mechanism that must be taken in consideration is the role of cytoskeletal-vesicle interaction in favoring or impeding exocytosis (Zajac et al., 2013). An alteration in the organization of cytoskeletal proteins could lead to an entrapping of the approaching vesicles thus overfavoring their collapse toward plasma membrane or even impeding their retrieval once fused. CoA/acetyl-CoA are important for a huge variety of intracellular reactions, including actin acetylation (Latario et al., 2020), and this could cause an aberrant organization of cytoskeletal proteins with consequent effects on vesicle exo-endocytosis as mentioned earlier (Zajac et al., 2013). When we tracked the fate of endosomes in living astrocytes, we found a significantly lower diffusion coefficient, a lower average distance, and a lower percentage of diffusive trajectories in motile endosomes from PKAN patients with respect to controls. Contrariwise, a significant increase in directed motions in PKAN astrocytes emerged. The alteration of these parameters further supports our hypothesis of an impediment to normal physiological intracellular trafficking of the endosomal compartment. Our data suggest both the presence of aberrant biophysical properties of endosomes, such as a different membranes lipid composition and an unbefitting cytoskeletal milieu. Moreover, an altered lipid composition by itself can cause problems in the interactions also between vesicles and normal cytoskeletal proteins. These circumstances are not exclusive but, on the contrary, could act synergistically in fueling the outcomes of PKAN phenotype. The general aberrant endosomal trafficking observed can be due to the combination of the intrinsic properties of the membranes and an alteration in the endosomes’ intracellular interactions. We tested the effect of treatment with two different compounds, CoA and 4-PBA, respectively, on the altered endosomal trafficking. Treatments with CoA have been demonstrated to be efficient in rescuing aberrant PKAN behavior and mitochondria functionality in hiPS-derived neurons (Orellana et al., 2016), and also in preventing iron deposition in > 70 days old hiPS-derived astrocytes (Santambrogio et al., 2022). We decided on direct treatments with CoA to confirm the efficacy of this molecule also in reverting endosomal aberrant trafficking in 50–60 days old astrocytes. Recently, a clear role of 4-PBA in maintaining proteostasis and relieving ER stress emerged (Kolb et al., 2015), suggesting multiple protective effects of this molecule from cellular stress. Moreover, 4-PBA treatment resulted in the reduction of phosphorylated E1α and increased PDHC activity both in mice and in fibroblasts from PDHC-deficient patients, and corrected morphological, locomotor, and biochemical abnormalities in the zebrafish model of PDHC deficiency (Ferriero and Brunetti-Pierri, 2013). It has been recently reported that PDHC enzymatic activity was significantly reduced in the globus pallidus of *Pank2* null mice (Jeong et al., 2019). This decrease in PDHC activity was hypothesized to arise from a decrease in lipoylation due to inactive mitochondrial Acyl Carrier Protein (mtACP) (Lambrechts et al., 2019). The active 4′-phosphopantetheinylated mtACP (holo-mtACP), plays a central role in the lipoic acid synthesis, used to modify mitochondrial proteins, among which is the E2 subunit of PDHC. In conditions of PANK2 depletion, levels of holo−mtACP were decreased in mammalian and *Drosophila* cells, and lipoylation of PDHC was also decreased. The impairment of CoA biosynthesis may lead to decreased levels of 4′-phosphopantetheinylated holo-mtACP, which causes decreased levels of lipoylated and activated PDHC (Lambrechts et al., 2019). Another evidence is the reduction of TfR1 palmitoylation, a post-translational regulation reported in different NBIA subtypes, which has been reverted by CoA treatment in *CRAT* and *PANK2* mutated cell lines (Drecourt et al., 2018). Altogether, these data suggest that boosting mitochondrial acetyl-CoA production by enhancing PDHC activity could impact the acetyl-CoA levels available not only in the mitochondria but also in the cytosol, which will be used in lipid metabolism necessary for the maintenance of membranes integrity and resulting in rescuing endosomes trafficking. In addition, recent evidence also showed the importance of acetyl-CoA in regulating organelles trafficking in the secretory pathway and the maintenance of proteostasis (Dieterich et al., 2021). Although with variable effects, both molecules were able to partially restore the normal endosomal activity, by re-establishing SB uptake and diffusion coefficient and trajectories toward control levels. The restoration of the normal exo-endocytosis activity also led to a significant decrease in Tf uptake and suggests that mitochondrial CoA defective biosynthesis in PKAN astrocytes could determine an overall impairment in endosomal behavior that ultimately trigger iron overload *via* TfR1-mediated intake. Moreover, the rescuing effect triggered by CoA and 4-PBA treatments on endosomes fate could also suggest that a potential defective mitochondrial acetyl-CoA/CoA metabolism is somehow sensed and feedback by different cytosolic players along the endosomal trafficking pathway. The iron overload and accumulation in astrocytes might activate a cascade of events, following iron-induced ROS generation, that sparks reactive gliosis. Our hypothesis is that the neurodegeneration seen in PKAN patients could happen also as a consequence of gliosis by excitotoxicity, due to aberrant exocytosis of toxic amounts of gliotransmitters, such as glutamate, as previously demonstrated in Santambrogio et al. (2022). It remains to clarify the potential role of iron deposition in the induction of reactive gliosis. However, our results have clearly demonstrated with a dedicated and sophisticated method, an impaired endosomal trafficking in PKAN astrocytes. Our study showed that CoA and 4-PBA treatments were able to revert the pathological phenotype without any attempts to investigate the mechanism of actions of the two molecules. Our results are observational and, considered together with established alterations found in PKAN, only allow to suggest hypothetic scenarios on how defective mitochondrial CoA homeostasis could impact on endosomal trafficking (Figure 5). Further studies to test our hypotheses and aimed to find a correlation between mitochondrial CoA homeostasis and endosomal fate are necessary to elucidate the mechanisms behind these behaviors.

## Data Availability Statement

The raw data supporting the conclusions of this article will be made available by the authors, without undue reservation.

## Author Contributions

MR performed pulse and chase experiments with Tf and SyanptoZip-Synbond, performed live tracking experiments with SynaptoZip-Synbond, collected data, and ran analyses. MR and PS cultured and maintained astrocytes for experiments. PS and AC performed Western blot experiments, collected data, and performed relative analyses. GR prepared SynaptoZip lentiviral vectors. IDM performed analyses of Tf uptake experiments in CoA and 4-PBA treated cells. MR, SL, and PS conceived this project, designed the experiments, and interpreted the data. MR, SL, and VT wrote and edited the manuscript. All authors contributed to the article and approved the submitted version.

## Conflict of Interest

The authors declare that the research was conducted in the absence of any commercial or financial relationships that could be construed as a potential conflict of interest.

## Publisher’s Note

All claims expressed in this article are solely those of the authors and do not necessarily represent those of their affiliated organizations, or those of the publisher, the editors and the reviewers. Any product that may be evaluated in this article, or claim that may be made by its manufacturer, is not guaranteed or endorsed by the publisher.

## Supplementary Material

Supplementary Figure 1 Characterization of astrocytes. Representative immunofluorescence images of control and PKAN[Gly420Valfs*30] astrocytes differentiated for 50 days. Astrocytes were stained with the specific marker glial fibrillary acidic protein (GFAP, green) and excitatory amino acid transporter 2 (EAAT2, red). Nuclei were stained with DAPI (scale bar 20 μm).

Supplementary Figure 2 TfR1 receptor expression and 5-min pulse transferrin uptake. **(A)** Blots showing TfR1 expression from *n* = 1 exemplar experiment. **(B)** Confocal exemplar images showing fluorescent transferrin (16 colors LUT as at the bottom, DAPI in Cyan) in Controls and PKAN astrocytes after 5 min pulse (scale bar 10 μm, *n* ≥ 103 cells analyzed for each, from *n* = 3 independent replicates). Error bars are SEM for all. **p* < 0.05; ^**^*p* < 0.01; ^***^*p* < 0.001; ^****^*p* < 0.0001 for all.

Supplementary Figure 3 5-min pulse Synbond uptake. **(A)** Confocal fluorescent exemplar images showing Synbond uptake (6 shades LUT as at the bottom, scale bar 20 μm), after 5 min pulse, in astrocytes expressing SynaptoZip (green) from control and PKAN astrocytes. **(B)** bars are mean vesicular fluorescence of Synbond in controls and PKAN endosomes (*t*-test, *n* ≥ 5,710 vesicles analyzed for each, from *n* = 3 independent replicates). **(C,D)** 2nd epoch SB mean vesicular fluorescence normalized as a percentage of 1st epoch SB mean vesicular fluorescence. (One-Way ANOVA, *n* ≥ 820 vesicles analyzed for each, from *n* = 3 independent replicates). **(E)** Time frames representing live binding dynamics in exemplar astrocytes (16 color LUT as at the bottom) expressing SynaptoZip (grayscale, scale bar 10 μm). Error bars are SEM for all. **p* < 0.05; ^**^*p* < 0.01; ^***^*p* < 0.001; ^****^*p* < 0.0001 for all.

Supplementary Figure 4 Effects of CoA and 4-PBA treatments on Transferrin and Synbond uptake. Exemplar confocal images from controls and PKAN astrocytes treated with CoA and 4-PBA, and relative uptake of fluorescent Transferrin (15 min pulse, middle panels, 16 color LUT as at the bottom) and Synbond (15 min pulse, right panels, 6 shades LUT as at the bottom; SynaptoZip fluorescence in green).

Supplementary Figure 5 Endosome recruitment. Exemplar confocal images from control **(A)** and PKAN astrocytes **(B)** expressing SynaptoZip (grayscale) showing endosomes and their relative SB uptake (5 and 15 min, LUT as at the bottom in untreated conditions or treated with CoA and 4-PBA.

Supplementary Movie 1 Exemplar Synbond-enriched endosome tracking showing a diffusive trajectory.

Supplementary Movie 2 Exemplar Synbond-enriched endosome tracking showing a directed trajectory.
