# Supplementary figures and images for: PKAN hiPS-Derived Astrocytes Show Impairment of Endosomal Trafficking: A Potential Mechanism Underlying Iron Accumulation
Source: Front Cell Neurosci. 2022 Jun 16;16:878103. doi: 10.3389/fncel.2022.878103 (PMC9243464; doi:10.3389/fncel.2022.878103)

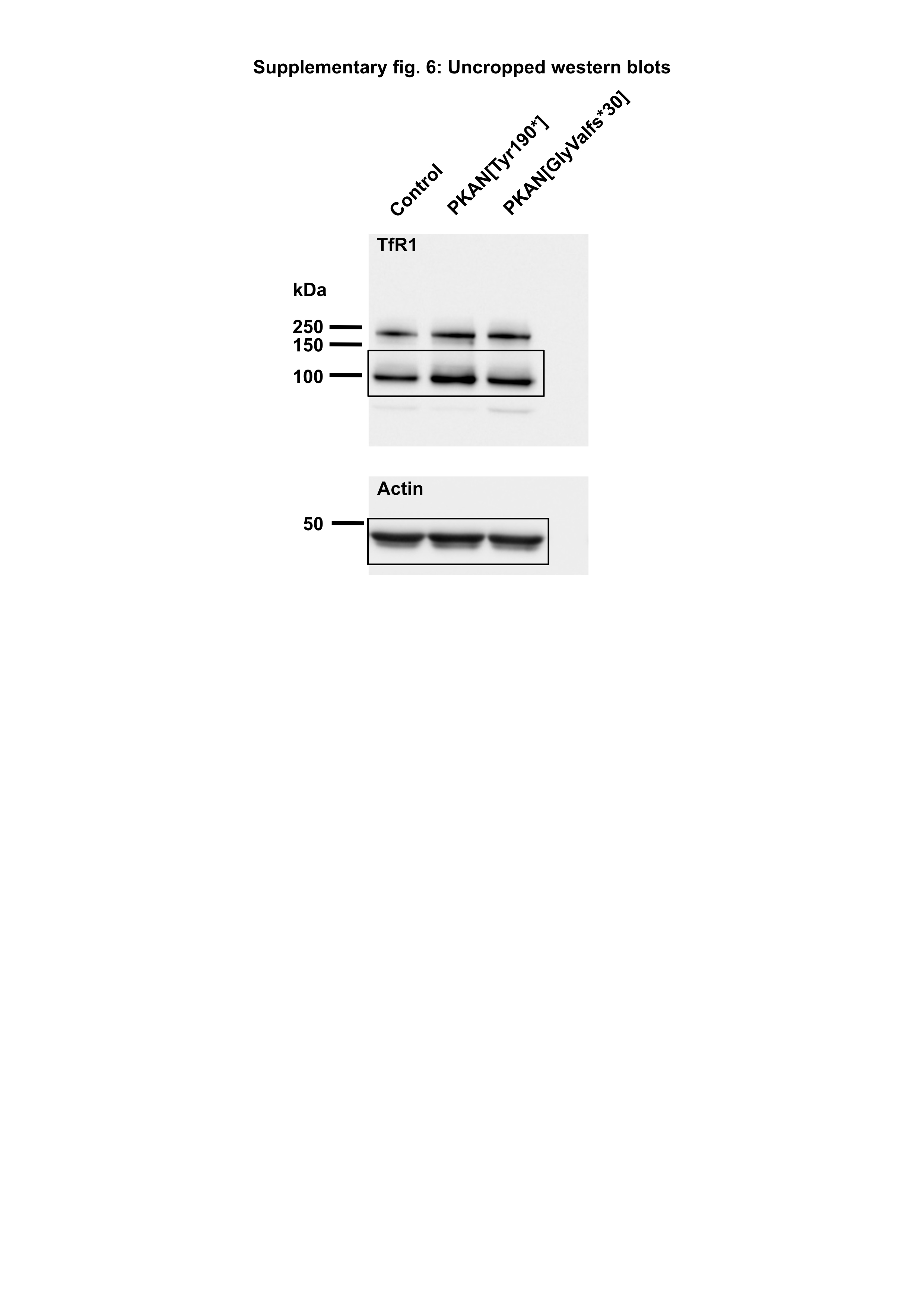

Supplement: Supplementary file 1 [file Image_6.JPEG]

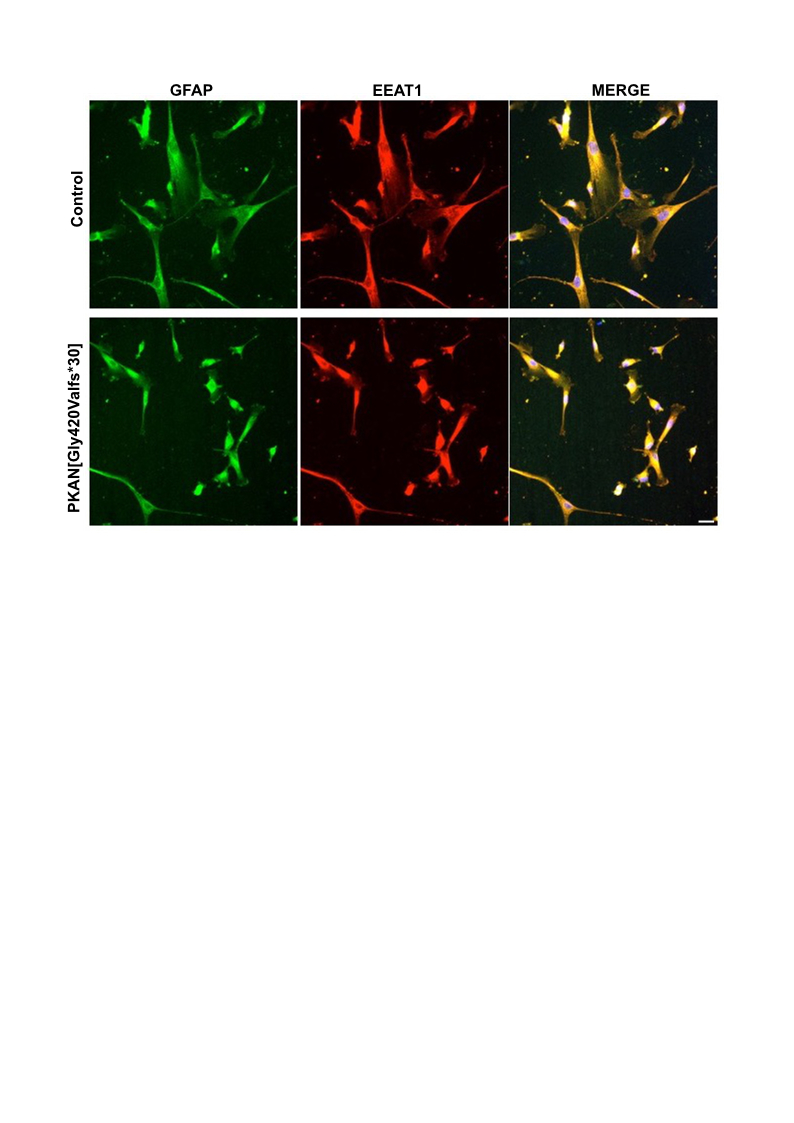

Supplement: Supplementary Figure 1 — Characterization of astrocytes. Representative immunofluorescence images of control and PKAN[Gly420Valfs*30] astrocytes differentiated for 50 days. Astrocytes were stained with the specific marker glial fibrillary acidic protein (GFAP, green) and excitatory amino acid transporter 2 (EAAT2, red). Nuclei were stained with DAPI (scale bar 20 μm). [file Image_1.JPEG]

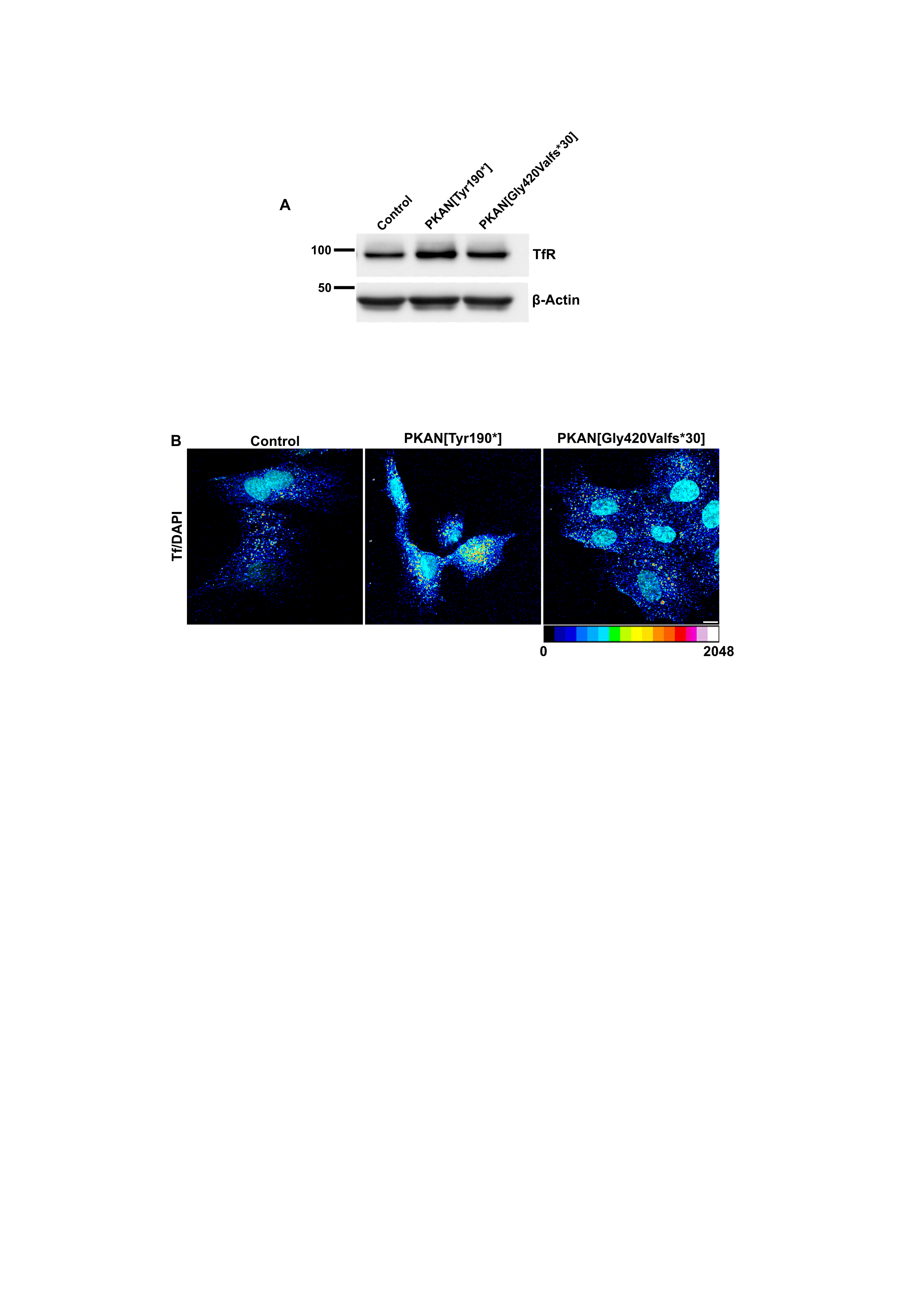

Supplement: Supplementary Figure 2 — TfR1 receptor expression and 5-min pulse transferrin uptake. (A) Blots showing TfR1 expression from n = 1 exemplar experiment. (B) Confocal exemplar images showing fluorescent transferrin (16 colors LUT as at the bottom, DAPI in Cyan) in Controls and PKAN astrocytes after 5 min pulse (scale bar 10 μm, n ≥ 103 cells analyzed for each, from n = 3 independent replicates). Error bars are SEM for all. *p < 0.05; **p < 0.01; ***p < 0.001; ****p < 0.0001 for all. [file Image_2.JPEG]

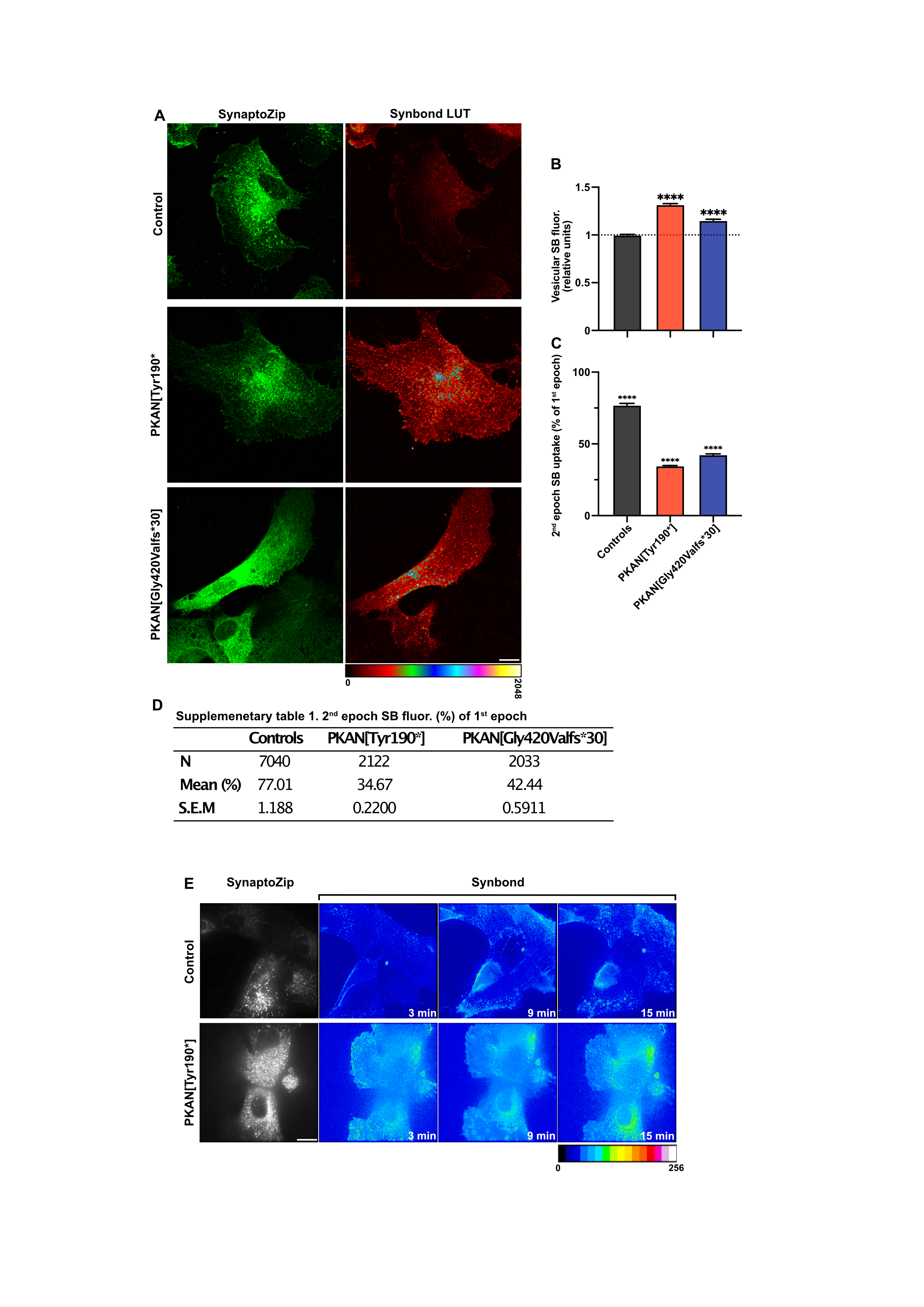

Supplement: Supplementary Figure 3 — 5-min pulse Synbond uptake. (A) Confocal fluorescent exemplar images showing Synbond uptake (6 shades LUT as at the bottom, scale bar 20 μm), after 5 min pulse, in astrocytes expressing SynaptoZip (green) from control and PKAN astrocytes. (B) bars are mean vesicular fluorescence of Synbond in controls and PKAN endosomes (t-test, n ≥ 5,710 vesicles analyzed for each, from n = 3 independent replicates). (C,D) 2nd epoch SB mean vesicular fluorescence normalized as a percentage of 1st epoch SB mean vesicular fluorescence. (One-Way ANOVA, n ≥ 820 vesicles analyzed for each, from n = 3 independent replicates). (E) Time frames representing live binding dynamics in exemplar astrocytes (16 color LUT as at the bottom) expressing SynaptoZip (grayscale, scale bar 10 μm). Error bars are SEM for all. *p < 0.05; **p < 0.01; ***p < 0.001; ****p < 0.0001 for all. [file Image_3.jpg]

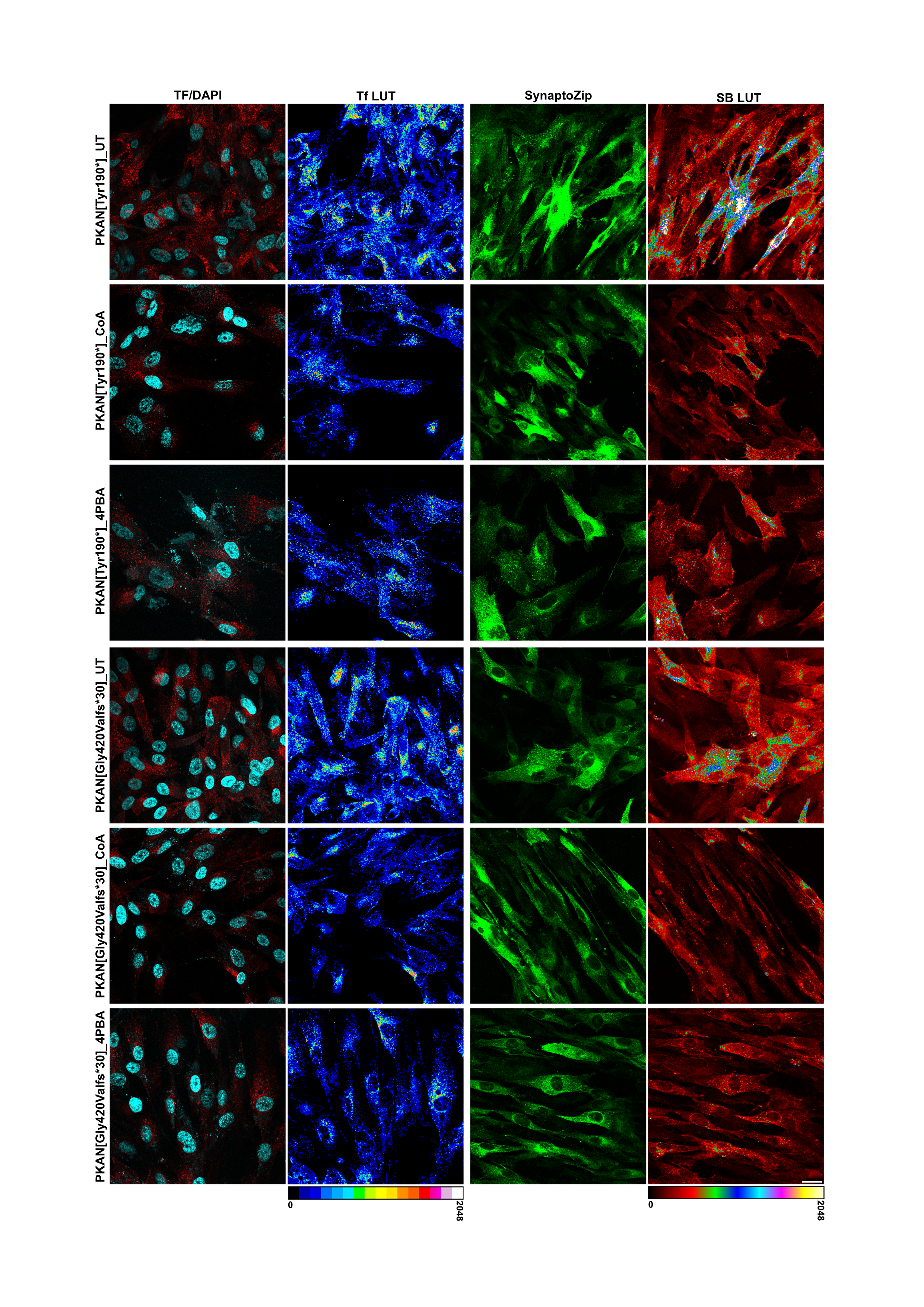

Supplement: Supplementary Figure 4 — Effects of CoA and 4-PBA treatments on Transferrin and Synbond uptake. Exemplar confocal images from controls and PKAN astrocytes treated with CoA and 4-PBA, and relative uptake of fluorescent Transferrin (15 min pulse, middle panels, 16 color LUT as at the bottom) and Synbond (15 min pulse, right panels, 6 shades LUT as at the bottom; SynaptoZip fluorescence in green). [file Image_4.JPEG]

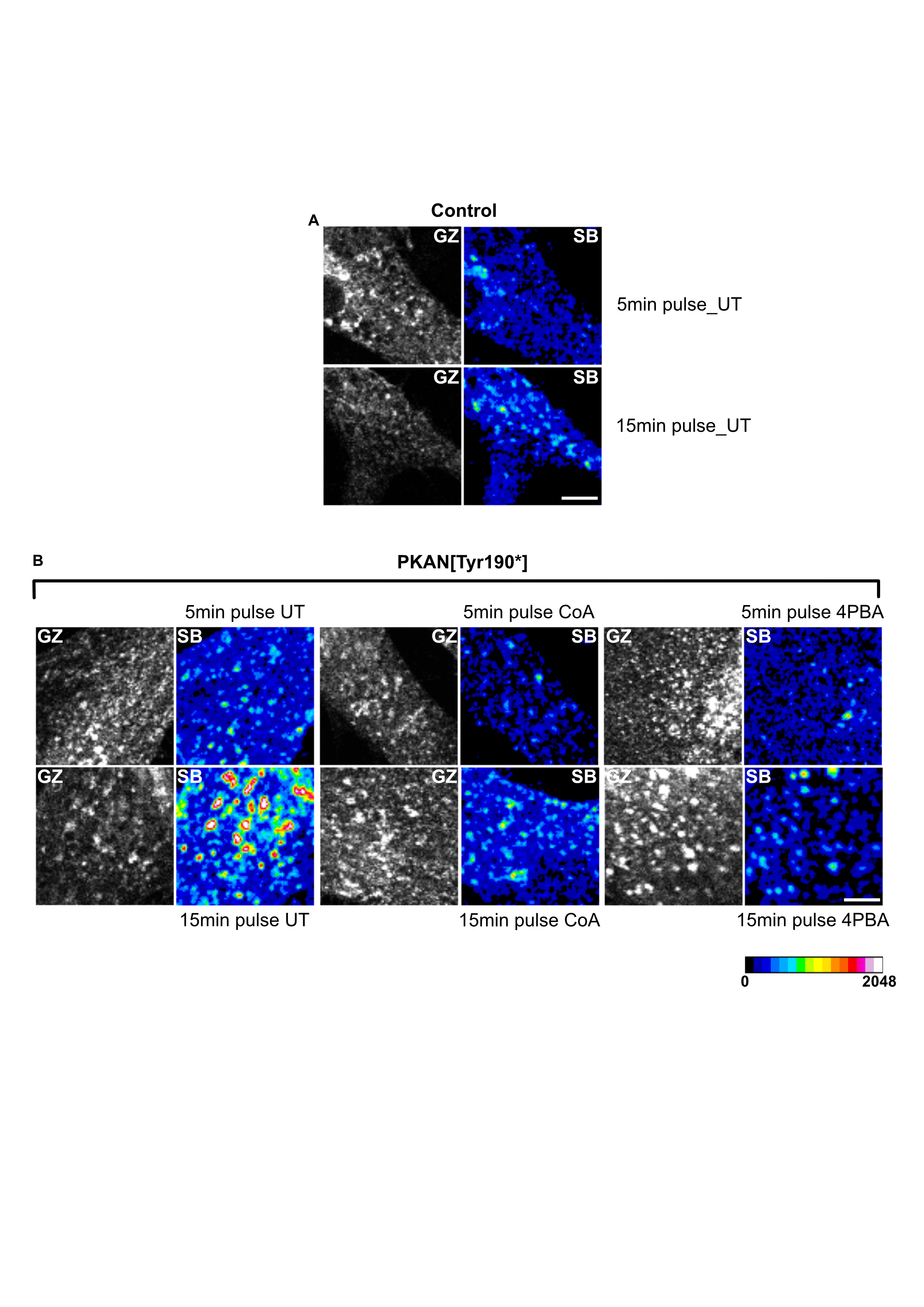

Supplement: Supplementary Figure 5 — Endosome recruitment. Exemplar confocal images from control (A) and PKAN astrocytes (B) expressing SynaptoZip (grayscale) showing endosomes and their relative SB uptake (5 and 15 min, LUT as at the bottom in untreated conditions or treated with CoA and 4-PBA. [file Image_5.JPEG]
